# Facial/sinus pain or pressure and migraine: exploratory findings from the HEADS registry

**DOI:** 10.3389/fpain.2025.1625442

**Published:** 2025-08-21

**Authors:** Deena E. Kuruvilla, Gretchen E. Tietjen, Gregory A. Panza, Victoria L. Hodgkinson, Frederick A. Godley

**Affiliations:** ^1^ Brain Health Institute, Westport, CT, United States; ^2^ Department of Neurology, University of Toledo, Toledo, OH, United States; ^3^ Department of Research, Hartford HealthCare, Hartford, CT, United States; ^4^ Lumiio Inc., Calgary, AB, Canada; ^5^ Department of Clinical Neurosciences, University of Calgary, Calgary, AB, Canada; ^6^ University Otolaryngology, Providence, RI, United States

**Keywords:** facial pain, migraine, rhinosinusitis, allergies, antibiotics, facial pressure, nasal congestion, rhinorrhea

## Abstract

**Background:**

Rhinosinusitis (RS) is a leading reason for antibiotic prescriptions but treatment satisfaction is low. Misdiagnosis may contribute to poor outcomes, as migraine—often underrecognized—can mimic RS symptoms, with studies showing overlap between RS and migraine diagnoses. Our aims were to explore the demographics and clinical features of facial pain or pressure (FPP), its relationship with migraine and RS, and distinguish symptoms between these overlapping conditions.

**Methods:**

The HEADS Registry, a web-based survey, targets adults with head and/or neck symptoms. Participants who answered “yes” (FPP+) or “no” (FPP−) to experiencing recurrent facial or sinus pain/pressure were included in this analysis. The ID Migraine screening tool was used to classify participants as ID Migraine+ or ID Migraine−. Demographics, symptoms, disability, history of allergies, sinusitis, and antibiotic use were compared between 1) FPP+ and FPP− groups, 2) FPP+/ ID Migraine+ and FPP+/ID Migraine−, and 3) FPP+/ID Migraine− and FPP−/ID Migraine+ subgroups. Continuous variables were compared using independent samples t-test or Mann–Whitney U, and categorical variables were compared using chi-square or Fisher's exact test.

**Results:**

The FPP+ group (*n* = 598) was younger, more often female, and reported higher rates of nasal, vestibular, and otologic symptoms compared to the FPP− group (*n* = 146). They also had more severe headaches, migraine-associated symptoms, and higher ID Migraine screening rates. The FPP+ group reported greater daily symptom interference, and more allergies, sinus infections, and antibiotic use. Those who screened positive for migraine (FPP+/ID Migraine+, *n* = 438) had more severe symptoms, greater disability, and more frequent forehead/eye pain. FPP+/ID Migraine− (*n* = 48) participants were more likely to report nasal symptoms, allergies, and sinus infections, while FPP−/ID Migraine+ (*n* = 85) participants reported more disabling headaches.

**Conclusion:**

In this exploratory analysis, FPP was strongly associated with headache, including migraine, as well as allergies, rhinosinusitis, and antibiotic use. The low reported effectiveness of antibiotics suggests potential misdiagnosis. Findings that migraine, plus autonomic, vestibular, otologic symptoms are associated with FPP, highlight the need to expand the differential diagnosis beyond infectious causes. These insights, along with ongoing registry improvements, will support efforts to refine diagnostic accuracy and optimize treatment strategies for neurologic, otologic, and rhinologic conditions.

## Introduction

Each year in the United States, nearly one in 10 adults is diagnosed with rhinosinusitis (RS), the acute form of which is characterized by either purulent nasal discharge accompanied by nasal obstruction, facial pain-pressure-fullness, or both (1). The majority of those diagnosed with RS report severe symptoms and substantial adverse impact on daily functioning, and it is among the most common diagnoses for which antibiotics are prescribed (2, 3). Despite the high per annum costs of RS ($22 billion, direct and indirect) (4), treatment effectiveness and satisfaction is low (5–9). This could be related to the appropriateness of the prescribed antibiotic for the underlying infection, but these findings also raise the possibility that a subset of persons not responding to RS treatment are misdiagnosed. One common condition, characterized by symptoms that may overlap with those of RS, is migraine. Migraine is poorly recognized by patients and also widely underdiagnosed and misdiagnosed by both general practitioners and specialists (10). According to a recent study, 60% of people diagnosed with migraine reported one or more rhinologic symptoms and 20% met the formal criteria for rhinosinusitis during migraine attacks (11). In a 2018 study of 1,458 random patients presenting to 14 different otolaryngology clinics, those who screened positively for migraine 89.1% reported facial pressure (12). Notably, clinical studies have also shown that the majority of people diagnosed with RS met diagnostic criteria for migraine. In addition, studies highlight symptom improvement in this population with migraine-specific treatments, as opposed to antibiotics and/or surgery (13–15).

Although it not unusual for common conditions like RS and migraine to co-occur, we hypothesize that in a subset of persons experiencing recurrent episodes of facial/sinus pain or pressure (FPP), there may be an underlying migraine-like neurogenic etiology distinct from the infectious etiology of rhinosinusitis. With the ultimate goal of improving diagnostic accuracy and management for migraine and RS in persons presenting with FPP, the initial aim of this exploratory study was to examine and describe the demographic and clinical characteristics associated with FPP, its relationship with migraine, defined by symptomatology, and relationship with RS history and treatment effectiveness. Our second aim was to examine differences in demographics and clinical characteristics between subgroups, namely (1) FPP with migraine vs. FPP without migraine, and (2) FPP without migraine vs. migraine without FPP, in order to identify symptoms at the intersection of these two overlapping conditions, and those that distinguish them. With an eye toward clinical applicability of our findings, we chose to study a cohort based on a self-reported symptom, facial/sinus pain or pressure, rather than on the diagnosis of a condition that presumes a specific temporal course [e.g., persistent idiopathic facial pain (16), anatomic origin [e.g., sinus headache, orofacial pain (17)] or pathophysiology (e.g., infectious, allergic, neurogenic)]. In this study migraine status was determined using a validated screening tool with a high positive predictive value (18). We utilized the recently established Headache, Ear, Auditory, Dizzy and Sinus (HEADS) registry, which contains information on demographics, clinical characteristics, comorbid conditions, and response to treatment. The long-term goals of the HEADS Registry are to refine clinical diagnostic and treatment guidelines for neurologic, otologic and rhinologic conditions, improve outcomes and advance medical education.

## Materials and methods

The HEADS Registry is a prospective, patient-reported set of questions (available in English and Spanish) deployed through a web-based portal. The registry is operated by the Association of Migraine Disorders (AMD) and registry data collection is IRB-approved [Advarra IRB, HEADS Registry (Pro0070553)].

### Participants

From July 2023 to September 2024, participants in the HEADS registry were recruited through a variety of means, including their medical professionals, AMD contact channels, social media, and partnership with nonprofit organizations, the Coalition for Headache and Migraine Patients (CHAMP) and Vestibular Disorders Association (VeDA). Recruitment into the registry was based on participants having head and/or neck symptoms, including headache, rhinosinusitis (chronic or recurring), and dizziness. Other criteria for registry participation included residence in the United States, aged 18 years or older, ability to understand English or Spanish, and signed the informed consent. This study included a subset of the registry responding either “yes” [FPP positive (FPP+)] or “no” [FPP negative (FPP−)] to the question, “Have you repeatedly experienced pain or pressure anywhere across your face or in your sinuses?”.

### Registry questionnaire

The HEADS registry questionnaire was developed from a trial survey completed by 1,631 participants, then refined for clarity and completeness by a team of otolaryngologists, neurologists, physical therapists, psychologists, and patient advocates. There are 12 sections of the survey consisting of 50 head and neck symptom-related questions (see Supplementary Material), however, for this analysis we focused on questions relevant to our specific aims. Branching logic was used to limit question burden where possible. All questions were optional. To identify individuals with high likelihood of migraine, the validated, self-reported ID Migraine screening tool was utilized. ID Migraine consists of three questions about headaches occurring during the prior three months involving (1) limitation of activities for at least one day in the past 3 months, (2) nausea or vomiting, and (3) light sensitivity. A positive screen is a “yes” answer to two or more of the questions and indicates a positive predictive value of migraine of 93% (18). Another validated tool, Migraine Disability Assessment (MIDAS), was used to determine headache-related disability (19).

### Statistical analysis methods

Data were assessed for normality and to ensure assumptions were met for the statistical tests conducted. Patient demographics, clinical symptoms, disabling features, allergy history and testing, sinusitis history, and related antibiotic use were compared based on FPP status, i.e., FPP+ group vs. FPP− group. We conducted subgroup analyses to further explore if there were differences between participants who screened positive for migraine using the three-item ID Migraine tool (ID Migraine+) vs. participants who screened negative (ID Migraine−) within the FPP+ group. We also compared the subgroup with FPP but no migraine (FPP+/ID Migraine−) to the subgroup with migraine but no FPP (FPP−/ID Migraine+) to explore differences when isolating FPP+ patients and ID Migraine+ patients. The subgroup analyses excluded participants who did not respond to the ID Migraine questions. Continuous variables were compared between groups using an independent samples *t*-test or Mann–Whitney *U* test depending on distribution of data, and presented as mean ± standard deviation or median, interquartile range, respectively. Categorical variables were compared using chi-square test or Fisher's exact test and presented as frequency (%). Bonferroni correction was used to adjust for multiple comparisons when appropriate. Statistical analysis was performed using Statistical Package for the Social Sciences, Version 29.0 (IBM Corp., Armonk, NY, USA) and *P* < .05 was established as the level of statistical significance.

## Results

### Study enrollment and population demographics

**Figure 1:**
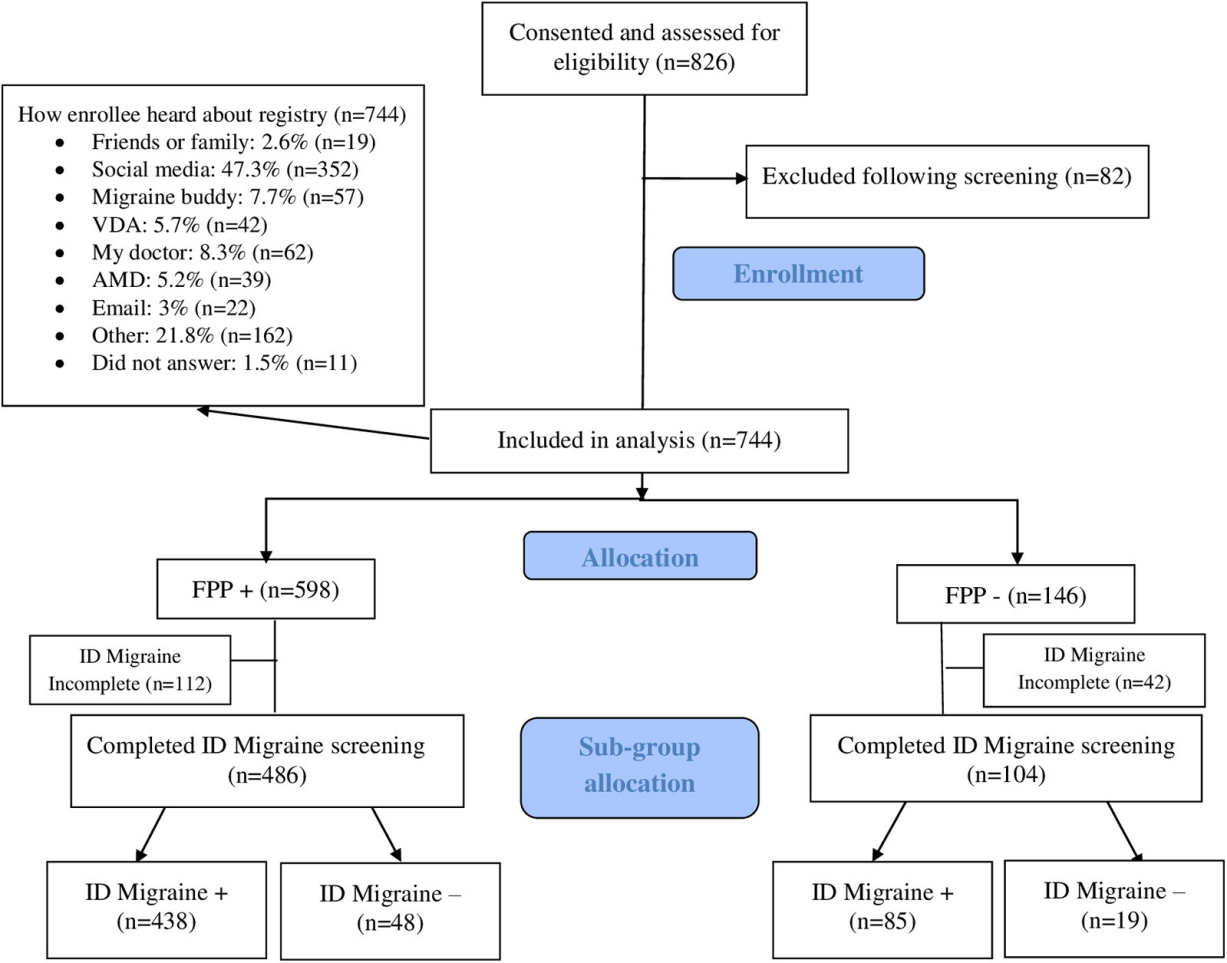
Flow diagram.

Over the 14 months of our study, a total of 826 participants with head and/or neck symptoms consented to participate in the HEADS registry (Figure 1, flow chart). After exclusion following screening, 744 participants were included, nearly half coming to the study through social media (Figure 1, flow chart). Of the 744 participants, 598 (80.4%) were FPP+, while 146 (19.6%) were FPP−. In the FPP+ group, 486 participants completed the ID Migraine screen, with 438 (90.1%) stratified to the ID Migraine+ subgroup, and 48 (9.9%) to the ID Migraine− subgroup. In the FPP− group, 104 participants completed the ID Migraine screen, with 85 (81.7%) stratified to the ID Migraine+ subgroup.

### Demographics, symptom variables, disabling features, allergies and sinusitis in FPP+ and FPP− groups

#### Demographics

Participants in the FPP+ group were younger in years (50.36 ± 13.59 vs. 53.67 ± 14.13, *P* = .012), and more likely to be female (93.5% vs. 86.3%, *P* = .011) than those in the FPP− group. There were no significant differences in race or ethnicity between groups and participants (FPP+ vs. FPP−) were predominantly White (90.3% vs. 86.3%) and non-Hispanic (96.5% vs. 96.4%; Table 1).

**Table 1:** Patient demographic and symptom characteristics: FPP+ vs. FPP−.

Variable	FPP+ (*n* = 598)	FPP− (*n* = 146)	Total (*N* = 744)	*P*
Demographics				
Age, yr (mean ± SD)	50.36 ± 13.59	53.67 ± 14.13	51.01 ± 13.75	.012
Sex				.011
Female	559 (93.5)*	126 (86.3)	685 (86.3)	
Male	37 (6.2)	18 (12.3)*	18 (12.3)	
Undeclared	2 (0.3)	2 (1.4)	4 (0.5)	
Race				.311
American Indian or Alaska Native	12 (2.0)	4 (2.7)	16 (2.2)	
Asian	6 (1.0)	3 (2.1)	9 (1.2)	
Black or African American	15 (2.5)	4 (2.7)	19 (2.6)	
Native Hawaiian or Other Pacific Islander	0 (0.0)	1 (0.7)	1 (0.1)	
White	540 (90.3)	126 (86.3)	666 (89.5)	
Other	14 (2.3)	3 (2.1)	17 (2.3)	
Undeclared	11 (1.8)	5 (3.4)	16 (2.2)	
Ethnicity				.729
Non-Hispanic	577 (96.5)	140 (95.9)	717 (96.4)	
Hispanic	21 (3.5)	6 (4.1)	27 (3.6)	
Symptom variables				
History of frequent nasal symptoms	449 (75.6)	57 (39.0)	506 (68.4)	<.001
History of dizziness	520 (87.5)	116 (80.6)	636 (86.2)	.029
History of auditory symptoms	538 (90.3)	117 (80.7)	655 (88.4)	.001
ID Migraine				.002
Yes	438 (73.2)*	85 (58.2)	523 (70.3)	
No	48 (8.0)	19 (13.0)	67 (9.0)	
Incomplete	112 (18.7)	42 (28.8)*	154 (20.7)	
Headache that limited activities for ≥1 day in last 3 months	*n* = 486	*n* = 104	*n* = 590	<.001
	453 (93.2)	84 (80.8)	537 (91.0)	
Headache symptom nausea/vomiting	353 (59.0)	67 (45.9)	420 (56.5)	.004
Headache symptom light sensitivity	422 (70.6)	82 (56.2)	504 (67.7)	<.001
History of frequent or severe headache	*n* = 486	*n* = 128	*n* = 636	<.001
	489 (96.3)	104 (81.3)	593 (93.2)	
Family history of migraine disorder or frequent headache	*n* = 508	*n* = 128	*n* = 636	<.001
	362 (71.4)	71 (55.5)	433 (68.2)	
Age when initial headache occurred, yr	*n* = 487	*n* = 103	*n* = 590	.429
<12	140 (28.7)	24 (23.3)	164 (27.8)	
12–25	219 (45.0)	45 (43.7)	264 (44.7)	
26–40	90 (18.5)	26 (25.2)	116 (19.7)	
41–65	34 (7.0)	8 (7.8)	42 (7.1)	
>65	4 (0.8)	0 (0.0)	4 (0.7)	
Nasal secretions description	*n* = 279	*n* = 29	*n* = 308	.854
Thin and clear	188 (67.4)	23 (79.3)	211 (68.5)	
Sometimes thick yellow	1 (0.4)	0 (0.0)	1 (0.3)	
Thick and discolored	38 (13.6)	2 (6.9)	40 (13.0)	
Thick and bad tasting	8 (2.9)	0 (0.0)	8 (2.6)	
Thick and white	42 (15.1)	4 (13.8)	46 (14.9)	
Thin, clear, and discolored bad tasting	1 (0.4)	0 (0.0)	1 (0.3)	
Thick, clear, and sometimes green	1 (0.4)	0 (0.0)	1 (0.3)	
Disabling features				
Symptoms interfere daily	577 (96.5)	131 (89.7)	708 (95.2)	<.001
FPP symptoms interfere daily	200 (33.4)	4 (2.7)	204 (27.4)	<.001
Dizziness symptoms interfere daily	256 (42.8)	60 (41.1)	316 (42.5)	.707
Ear symptoms interfere daily	116 (19.4)	22 (15.1)	138 (18.5)	.228
Headache symptoms interfere daily	424 (70.9)	75 (51.4)	499 (67.1)	<.001
Most disabling symptom	*n* = 427	*n* = 94	*n* = 521	<.001
Dizziness	76 (17.8)	33 (35.1)*	109 (20.9)	
Ear	8 (1.9)	8 (8.5)*	16 (3.1)	
FPP	33 (7.7)*	0 (0.0)	33 (6.3)	
Headache	295 (69.1)*	52 (55.3)	347 (66.6)	
Unknown	15 (3.5)	1 (1.1)	16 (3.1)	
MIDAS grade for headache	*n* *=* 334	*n* *=* 61	*n* = 395	.783
I	13 (13.9)	3 (4.9)	16 (4.1)	
II	12 (3.6)	3 (4.9)	15 (3.8)	
III	43 (12.9)	10 (16.4)	53 (13.4)	
IV	266 (79.6)	45 (73.8)	311 (78.7)	
MIDAS score for headache (median, IQR)	*n* = 334	*n* = 61	*n* = 395	.119
	66.5, 104.0	45.0, 82.5	60.0, 101.0	
Allergies and sinusitis				
Allergies	386 (64.5)	54 (37.0)	440 (59.1)	<.001
Allergy testing	283 (73.9)	33 (61.1)	316 (72.3)	.049
Kind of allergy				
Trees, grass, weeds, pollen	222 (37.1)	24 (16.4)	246 (33.1)	<.001
Animal dander	151 (25.3)	15 (10.3)	166 (22.3)	<.001
Dust mites	186 (31.1)	23 (15.8)	209 (28.1)	<.001
Mold	163 (27.3)	16 (11.0)	179 (24.1)	<.001
Other	86 (14.4)	6 (4.1)	92 (12.4)	<.001
Not sure	7 (1.2)	0 (0.0)	7 (0.9)	.356
Sinus infection of sinusitis	276 (46.2)	19 (13.0)	295 (39.7)	<.001
Sinus infection lifetime frequency	*n* = 273	*n* = 19	*n* = 292	.253
1–3	25 (9.2)	4 (21.1)	29 (9.9)	
4–6	38 (13.9)	4 (21.1)	42 (14.4)	
7–9	46 (16.8)	2 (20.5)	48 (16.4)	
>10	164 (60.1)	9 (47.4)	173 (59.2)	
Antibiotics				
Antibiotic use	*n* = 387	*n* = 106	*n* = 493	<.001
	102 (26.4)	10 (9.4)	112 (22.7)	
Antibiotic indication	*n* = 69	*n* = 6	*n* = 75	.914
Preventive	2 (2.9)	0 (0.0)	2 (2.7)	
Acute	56 (81.2)	5 (83.3)	61 (81.3)	
Other	11 (15.9)	1 (16.7)	12 (16.0)	
Antibiotic effectiveness	*n* = 70	*n* = 6	*n* = 76	.797
Not effective	13 (18.6)	2 (33.3)	15 (19.7)	
Minimally effective	9 (12.9)	1 (16.7)	10 (13.2)	
Moderately effective	20 (28.6)	1 (16.7)	21 (27.6)	
Extremely effective	28 (40.0)	2 (33.3)	30 (39.5)	
Antibiotic effective for FPP	*n* = 102	*n* = 10	*n* = 112	.085
	38 (37.3)	1 (10.0)	39 (34.8)	
Antibiotic frequency	*n* = 66	*n* = 5	*n* = 71	.579
Less than 1 day per month	54 (81.8)	5 (100.0)	59 (83.1)	
Less than 15 days per month	7 (10.6)	0 (0.0)	7 (9.9)	
More than 15 days per month	5 (7.6)	0 (0.0)	5 (7.0)	
Antibiotic duration	*n* = 66	*n* = 5	*n* = 71	.657
Less than 1 month	39 (59.1)	2 (40.0)	41 (57.7)	
1–3 months	2 (3.0)	0 (0.0)	2 (2.8)	
3–12 months	3 (4.5)	0 (0.0)	3 (4.2)	
More than 1 year	22 (33.3)	3 (60.0)	25 (35.2)	

Categorical variables presented as frequency (%). Denominators are depicted in the top row of the table unless otherwise shown within the variable row. Variables with multiple comparisons adjusted using Bonferroni correction.

* Depicts where statistical significance occurred after Bonferroni adjustment. Fisher's exact test is used for variables with cell counts less than 5.

#### Symptom variables

Participants in the FPP+ group reported a greater rate of frequent nasal symptoms (e.g., runny nose, or nasal congestion) than the FPP− group (75.6% vs. 39.0%, *P* < .001) but no significant differences in nature of nasal secretions (Table 1). History of vestibular (e.g., dizziness, feeling off balance or having a spinning sensation) and otologic symptoms (e.g., ringing in ears, ear pressure, ear pain, sensation of blocked ears, or difficulty hearing) were also more common in FPP+ participants. A history of frequent or severe headache was common in the total sample (93.2%), but prevalence was higher in the FPP+ group (96.3% vs. 81.3%, *P* < .001). Participants in the FPP+ group also reported more migraine-associated symptoms with headache, including nausea/vomiting (59.0% vs. 45.9%, *P* = .004), sensitivity to light (70.6% vs. 56.2%, *P* < .001), and limitation of activities (93.2% vs. 80.8%, *P* < .001). The proportion with a positive ID Migraine screen was higher in the FPP+ group (73.2% vs. 58.2%, *P* = .002). In addition, FPP+ participants were more likely to have a family history of migraine or frequent headache (71.4% vs. 55.5%, *P* < .001).

#### Disabling features

The FPP+ group reported greater rates of daily interference for any symptom in general (96.5% vs. 89.7%, *P* < .001), and specifically those of FPP (33.4% vs. 2.7%, *P* < .001) and headache (70.9% vs. 51.4%, *P* < .001) (Table 1). Participants in the FPP+ group more commonly reported headache as their most disabling symptom (69.1% vs. 55.3%, *P* < .05 after Bonferroni adjustment), whereas a greater proportion of FPP− participants reported vestibular (35.1% vs. 17.8%, *P* < .05 after Bonferroni adjustment) and/or otologic symptoms (8.5% vs. 1.9%, *P* < .05 after Bonferroni adjustment) as their most disabling symptoms. There were no statistically significant differences between groups for MIDAS score or grade.

#### Allergies and sinusitis

The FPP+ participants reported greater rates of allergies (64.5% vs. 37.0%, *P* < .001), allergy testing (73.9% vs. 61.1%, *P* = .049), sinus infection or sinusitis (46.2% vs. 13.0%, *P* < .001), and antibiotic use for FPP symptoms (26.4 vs. 9.4%, *P* < .001) than FPP− participants (Table 1). There were, however, no significant differences between groups for lifetime frequency of sinus infection, nor for antibiotic indication, effectiveness, frequency, or duration of antibiotic use.

### Demographics, symptoms, disabling features, allergies and sinusitis in FPP+ group's ID Migraine+ and ID Migraine− subgroups

Results from the sub-group analysis examining demographics, symptoms, disabling features, and allergies and sinusitis between ID Migraine+ (*n* = 438) vs. ID Migraine− (*n* = 48) among FPP+ participants (*n* = 486) are shown in Table 2.

**Table 2:** Patient demographics and symptom characteristics between patients who are FPP+ and migraine ID+ vs. patients who are FPP+ and Migraine ID−.

Variable	Migraine ID+ (*n* = 438)	Migraine ID−(*n* = 48)	Total (*N* = 486)	*P*
Demographics				
Age, yr (mean ± SD)	49.42 ± 12.97	60.57 ± 11.46	50.53 ± 13.25	<.001
Sex				
Female	416 (95.0)*	40 (83.3)	456 (93.8)	.003
Male	20 (4.6)	8 (16.7)*	28 (5.8)	
Undeclared	2 (0.5)	0 (0.0)	2 (0.4)	
Race				.761
American Indian or Alaska Native	7 (1.6)	2 (4.2)	9 (1.9)	
Asian	5 (1.1)	0 (0.0)	5 (1.0)	
Black or African American	11 (2.5)	2 (4.2)	13 (2.7)	
Native Hawaiian or Other Pacific Islander	0 (0.0)	0 (0.0)	0 (0.0)	
White	397 (90.6)	42 (87.5)	439 (90.3)	
Other	10 (2.3)	1 (2.1)	11 (2.3)	
Undeclared	8 (1.8)	1 (2.1)	9 (1.9)	
Ethnicity				.178
Non-Hispanic	422 (96.3)	48 (100.0)	470 (96.7)	
Hispanic	16 (3.7)	0 (0.0)	16 (3.3)	
Symptoms				
History				
History of frequent nasal symptoms	331 (75.7)	41 (85.4)	372 (76.7)	.132
History of dizziness	38 (79.2)	382 (88.0)	420 (87.1)	.082
History of auditory symptoms	41 (85.4)	395 (90.4)	436 (89.9)	.278
Symptoms when having FPP				
Dizziness	263 (60.0)	20 (41.7)	283 (58.2)	.014
Tinnitus	262 (59.8)	30 (62.5)	292 (60.1)	.719
Tearing	192 (43.8)	15 (31.3)	207 (42.6)	.094
Red eyes	66 (15.1)	6 (12.5)	72 (14.8)	.634
Swelling of an eyelid	56 (12.8)	2 (4.2)	58 (11.9)	.099
Temporary eyelid drooping	54 (12.3)	7 (14.6)	61 (12.6)	.654
Voice changes	85 (19.4)	8 (16.7)	93 (19.1)	.647
Temporary speech loss	61 (13.9)	5 (10.4)	66 (13.6)	.500
Temporary double vision	60 (13.7)	2 (4.2)	62 (12.8)	.068
Unexplained tooth pain	186 (42.5)	12 (25.0)	198 (40.7)	.019
Rotten, smoky, other smell	118 (26.9)	7 (14.6)	125 (25.7)	.063
Burning sensation in mouth or tongue	39 (8.9)	3 (6.3)	42 (8.6)	.534
Nausea or vomiting	223 (50.9)	8 (16.7)	231 (47.5)	<.001
Face sweating or flushing	123 (28.1)	9 (18.8)	132 (27.2)	.168
Temporomandibular joint pain	175 (40.0)	12 (25.0)	187 (38.5)	.043
Wheezing	28 (6.4)	5 (10.4)	33 (6.8)	.293
Trouble breathing through nose	185 (42.2)	20 (41.7)	205 (42.2)	.939
Nasal congestion	274 (62.6)	29 (60.4)	303 (62.3)	.771
Runny or drippy nose	215 (49.1)	25 (52.1)	240 (49.4)	.693
Photophobia	323 (73.7)	17 (35.4)	340 (70.0)	<.001
Phonophobia	289 (66.0)	19 (39.6)	308 (63.4)	<.001
Smell sensitivity	225 (51.4)	10 (20.8)	235 (48.4)	<.001
Blocked ear or pressure in ear	233 (53.2)	22 (45.8)	255 (52.5)	.332
Difficulty hearing	105 (24.0)	11 (22.9)	116 (23.9)	.871
Headache	389 (88.8)	39 (81.3)	428 (88.1)	.125
Sore throat	116 (26.5)	11 (22.9)	127 (26.1)	.593
Postnasal drip sensation	232 (53.0)	26 (54.2)	258 (53.1)	.874
Cough	104 (23.7)	13 (27.1)	117 (24.1)	.607
Sneezing	94 (21.5)	15 (31.3)	109 (22.4)	.123
Itchy eyes, nose, or throat	145 (33.1)	16 (33.3)	161 (33.1)	.975
Other symptoms	30 (6.8)	2 (4.2)	32 (6.6)	.758
Symptom details				
Age when initial headache occurred, yr	*n* = 436	*n* = 48	*n* = 484	.002
Before 12	135 (31.0)*	4 (8.3)	139 (28.7)	
12–25	196 (45.0)	22 (45.8)	218 (45.0)	
26–40	73 (16.7)	16 (33.3)*	89 (18.4)	
41–65	28 (6.4)	6 (12.5)	34 (7.0)	
>65	4 (0.9)	0 (0.0)	4 (0.8)	
Age at symptom initiation, yr	*n* = 432	*n* = 46	*n* = 478	<.001
Before 12	140 (32.4)*	4 (8.7)	144 (30.1)	
12–25	173 (40.0)	15 (32.6)	188 (39.8)	
26–40	75 (17.4)	15 (32.6)*	90 (18.8)	
41–65	40 (9.3)	11 (23.9)*	51 (10.7)	
>65	4 (0.9)	1 (2.2)	5 (1.0)	
Age when symptoms were most severe, yr	*n* = 431	*n* = 46	*n* = 477	<.001
Before 12	13 (3.0)	2 (4.3)	15 (3.1)	
12–25	89 (20.6)	4 (8.7)	93 (19.5)	
26–40	177 (41.1)	13 (28.3)	190 (39.8)	
41–65	141 (32.7)	21 (45.7)	162 (34.0)	
>65	11 (2.6)	6 (13.0)*	17 (3.6)	
Symptom area				
Left cheek	187 (42.7)	22 (45.8)	209 (43.0)	.677
Right cheek	202 (46.1)	19 (39.6)	221 (45.5)	.388
Lateral cheek				.801
None	184 (42.0)	22 (45.8)	206 (42.4)	
Unilateral	119 (27.2)	11 (22.9)	130 (26.7)	
Bilateral	135 (30.8)	15 (31.3)	150 (30.9)	
Forehead	284 (64.8)	24 (50.0)	308 (63.4)	.043
Nose	163 (37.2)	19 (39.6)	182 (37.4)	.748
Eyes	340 (77.6)	28 (58.3)	368 (75.7)	.003
Other	71 (16.2)	5 (10.4)	76 (15.6)	.294
Nasal secretions	*n* = 214	*n* = 25	*n* = 239	.950
Thin and clear	141 (65.9)	19 (76.0)	180 (66.9)	
Sometimes thick yellow	1 (0.5)	0 (0.0)	1 (0.4)	
Thick and discolored	29 (13.6)	3 (12.0)	32 (13.4)	
Thick and bad tasting	5 (2.3)	0 (0.0)	5 (2.1)	
Thick and white	36 (16.8)	3 (12.0)	39 (16.3)	
Thin, clear, and discolored bad tasting	1 (0.5)	0 (0.0)	1 (0.4)	
Thick, clear, and sometimes green	1 (0.5)	0 (0.0)	1 (0.4)	
Excessive nasal secretions/snot/mucus	*n* = 215	*n* = 25	*n* = 240	.579
With or without FPP	152 (70.7)	19 (76.0)	171 (71.3)	
Only with FPP	63 (29.3)	6 (24.0)	69 (28.7)	
Symptom duration	*n* = 434	*n* = 47	*n* = 481	.544
<4 h	40 (9.2)	2 (4.3)	42 (8.7)	
4–72 h	203 (46.8)	23 (48.9)	226 (47.0)	
4–7 d	51 (11.8)	5 (10.6)	56 (11.6)	
8–14 d	19 (4.4)	3 (6.4)	22 (4.6)	
>2 wks	30 (6.9)	1 (2.1)	31 (6.4)	
>12 wks	91 (21.0)	13 (27.7)	104 (21.6)	
Symptom frequency, days per month	*n* = 434	*n* = 47	*n* = 481	.810
1–5	110 (25.3)	12 (25.5)	122 (25.4)	
6–10	76 (17.5)	6 (12.8)	82 (17.0)	
11–15	56 (12.9)	7 (14.9)	63 (13.1)	
16–20	59 (13.6)	7 (14.9)	66 (13.7)	
21–25	30 (6.9)	2 (4.3)	32 (6.7)	
26–31	22 (5.1)	1 (2.1)	23 (4.8)	
Constant	81 (1.7)	12 (25.5)	93 (19.3)	
Symptoms described as sinus headache	*n* = 433	*n* = 46	*n* = 479	.898
	249 (57.5)	26 (56.5)	275 (57.4)	
Headaches that feel different than FPP	*n* = 434	*n* = 47	*n* = 481	<.001
	425 (97.9)	38 (80.9)	463 (96.3)	
Headache concurrent with symptoms timing	*n* = 369	*n* = 32	*n* = 401	.369
Before	63 (17.1)	6 (18.8)	69 (17.2)	
After	117 (31.7)	6 (18.8)	123 (30.7)	
Same time	86 (23.3)	11 (34.4)	97 (24.2)	
Different time	103 (27.9)	9 (28.1)	112 (27.9)	
Headache same day as symptoms frequency	*n* = 369	*n* = 32	*n* = 401	.590
Never	3 (0.8)	0 (0.0)	3 (0.7)	
Rarely	27 (7.3)	3 (9.4)	30 (7.5)	
Sometimes	106 (28.7)	13 (40.6)	119 (29.7)	
Often	158 (42.8)	10 (31.3)	168 (41.9)	
Always	75 (20.3)	6 (18.8)	81 (20.2)	
FPP compared to headache, symptom severity	*n* = 371	*n* = 32	*n* = 403	.006
Always less severe	95 (25.6)	7 (21.9)	102 (25.3)	
Sometimes less severe	123 (33.2)	10 (31.3)	133 (33.0)	
Often less severe	142 (38.3)	10 (31.3)	152 (37.7)	
Always more severe	11 (3.0)	5 (15.6)*	16 (4.0)	
FPP compared to headache, symptom duration	*n* = 369	*n* = 32	*n* = 401	.002
Always shorter	112 (30.4)	8 (25.0)	120 (29.9)	
Sometimes lasts longer	152 (41.2)	7 (21.9)	159 (39.7)	
Often lasts longer	57 (15.4)	5 (15.6)	62 (15.5)	
Always lasts longer	48 (13.0)	12 (37.5)*	60 (15.0)	
Disabling features				
Symptoms interfere daily	427 (97.5)	42 (87.5)	17 (3.5)	<.001
FPP symptoms interfere daily	172 (39.3)	22 (45.8)	194 (39.9)	.378
Dizziness symptoms interfere daily	224 (51.1)	16 (33.3)	240 (49.4)	.019
Ear symptoms interfere daily	91 (20.8)	14 (29.2)	105 (21.6)	.180
Headache symptoms interfere daily	388 (88.6)	26 (54.2)	414 (85.2)	<.001
Most disabling symptom	*n* = 371	*n* = 35	*n* = 406	<.001
Dizziness	57 (15.4)	8 (22.9)	65 (16.0)	
Ear	2 (0.5)	2 (5.7)*	4 (1.0)	
FPP	23 (6.2)	9 (25.7)*	32 (7.9)	
Headache	275 (74.1)	15 (42.9)*	290 (71.4)	
Unknown	14 (3.8)	1 (2.9)	15 (3.7)	
MIDAS grade for headache	*n* = 306	*n* = 20	*n* = 326	<.001
I	7 (2.3)	4 (20.0)	11 (3.4)	
II	11 (3.6)	1 (5.0)	12 (3.7)	
III	36 (11.8)	5 (25.0)	41 (12.6)	
IV	252 (82.4)*	10 (50.0)	262 (80.4)	
MIDAS score for headache (median, IQR)	*n* = 306	*n* = 20	*n* = 326	.005
	70.0, 101.5	22.0, 86.8	67.5, 104.0	
Allergies and sinusitis				
Allergies	291 (66.4)	30 (62.5)	321 (66.0)	.584
Allergy testing	*n* = 289	*n* = 29	*n* = 318	.004
	221 (76.5)	15 (51.7)	236 (74.2)	
Kind of allergy				
Trees, grass, weeds, pollen	175 (40.0)	13 (27.1)	188 (38.7)	.082
Animal dander	121 (27.6)	10 (20.8)	131 (27.0)	.314
Dust mites	147 (33.6)	11 (22.9)	158 (32.5)	.135
Mold	137 (31.3)	11 (22.9)	148 (30.5)	.232
Other	71 (16.2)	3 (6.3)	74 (15.2)	.088
Not sure	5 (1.1)	1 (2.1)	6 (1.2)	.466
Sinus infection of sinusitis	212 (45.8)	22 (48.4)	234 (48.1)	.735
Sinus infection lifetime frequency				.088
1–3	18 (8.6)	3 (13.6)	21 (9.1)	
4–6	25 (12.0)	5 (22.7)	30 (13.0)	
7–9	32 (15.3)	6 (27.3)	38 (16.5)	
>10	134 (64.1)	8 (36.4)	142 (61.5)	
Antibiotics				
Antibiotic use	*n* = 314	*n* = 38	*n* = 352	.086
	91 (29.0)	6 (15.8)	97 (27.6)	
Antibiotic indication	*n* = 63	*n* = 4	*n* = 67	.658
Preventive	2 (3.2)	0 (0.0)	2 (3.0)	
Acute	52 (82.5)	4 (100.0)	56 (83.6)	
Other	9 (14.3)	0 (0.0)	9 (13.4)	
Antibiotic effectiveness	*n* = 63	*n* = 5	*n* = 68	.802
Not effective	12 (19.0)	1 (20.0)	13 (19.1)	
Minimally effective	9 (14.3)	0 (0.0)	9 (13.2)	
Moderately effective	17 (27.0)	2 (40.0)	19 (27.9)	
Extremely effective	25 (39.7)	2 (40.0)	27 (39.7)	
Antibiotic effective for FPP	*n* = 91	*n* = 6	*n* = 97	.124
	32 (35.2)	4 (66.7)	36 (37.11)	
Antibiotic frequency	*n* = 60	*n* = 5	*n* = 65	.406
Less than 1 day per month	50 (83.3)	3 (60.0)	53 (81.5)	
Less than 15 days per month	6 (10.0)	1 (20.0)	7 (10.8)	
More than 15 days per month	4 (6.7)	1 (20.0)	5 (7.7)	
Antibiotic duration	*n* = 60	*n* = 5	*n* = 65	.923
Less than 1 month	35 (58.3)	3 (60.0)	38 (58.5)	
1–3 months	2 (3.3)	0 (0.0)	2 (3.1)	
3–12 months	3 (5.0)	0 (0.0)	3 (4.6)	
More than 1 year	20 (33.3)	2 (40.0)	22 (33.8)	

Categorical variables presented as frequency (%). Denominators are depicted in the top row of the table unless otherwise shown within the variable row. Variables with multiple comparisons adjusted using Bonferroni correction.

* Depicts where statistical significance occurred after Bonferroni adjustment. Fisher's exact test used for variables with cell counts less than 5.

#### Demographics

Participants in the ID Migraine+ subgroup were younger in years (49.42 ± 12.97 vs. 60.57 ± 11.46, *P* < .001), and more likely to be female (95.0% vs. 83.3%, *P* = .003) than the ID Migraine− subgroup. There were no differences in race and ethnicity between subgroups.

#### Symptom variables

History of frequent nasal symptoms, vestibular symptoms and otologic symptoms exceeded 75% in each of the FPP+ ID Migraine subgroups, and there were no significant differences. Participants were asked to select all that apply from a list of 30 symptoms involving the face, head, sinuses, nose, eyes, ears, mouth, throat, and respiratory tract in response to the question, “When you have facial/sinus pain or pressure, what are the symptoms that you experience?”. Headache was the most commonly reported symptom during FPP symptoms, but there were no significant differences between ID Migraine+ and ID Migraine− subgroups for this variable (88.8% vs. 81.3%, *P* = .125). A number of migraine-associated symptoms were, however, more frequently reported by the ID Migraine+ participants, including photophobia, phonophobia, osmophobia, nausea or vomiting, and dizziness. Temporomandibular joint pain and unexplained tooth pain were also more common in the ID Migraine+ subgroup. There were no significant differences between ID Migraine+ subgroups for any other symptoms, including those commonly described in persons with allergies and rhinosinusitis, although the larger proportion of eye tearing, eyelid swelling, and malodorous (rotten, smoky) smell in the ID Migraine+ subgroup approached significance and may warrant continued observation as registry recruitment increases.

#### Symptom details

**Figure 2:**
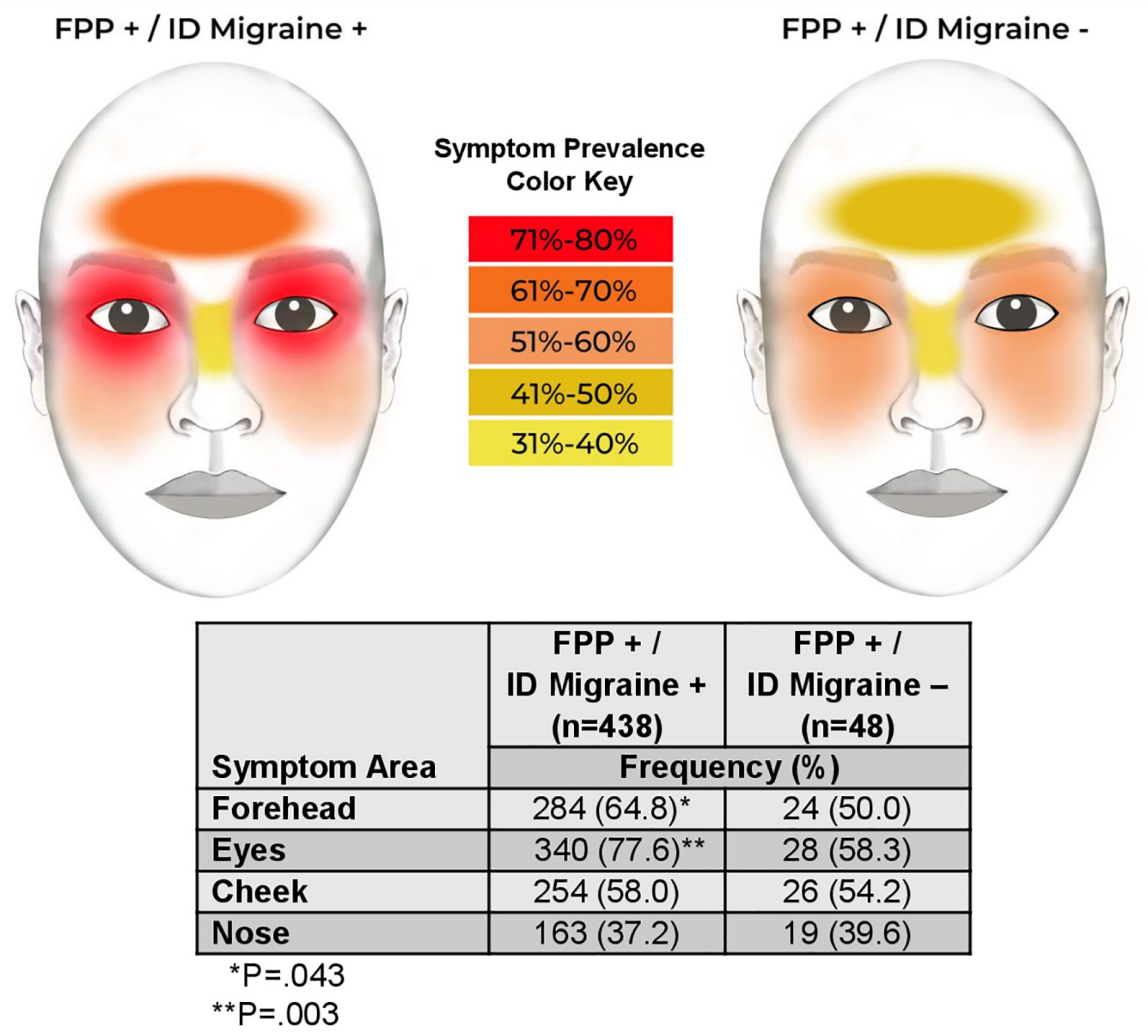
The prevalence of reported symptoms for each area of the face by ID migraine group.

Both headache and FPP symptoms were more likely to start before age 12 years in the ID Migraine+ subgroup, and at a later age in the ID Migraine-subgroup. Furthermore, ID Migraine+ participants were more likely to report their most severe symptoms at an earlier age, whereas they occurred after age 65 years in the ID Migraine− participants. Pain or pressure location was also different between subgroups, occurring more frequently over the forehead and eyes in the ID Migraine+ participants (Figure 2). There were no differences in the nature of nasal secretions between subgroups or whether excessive secretions occurred only during FPP symptoms or at other times as well. In addition, FPP symptom duration and frequency did not differ based on ID Migraine status. In response to questions addressing the interface of FPP symptoms and headache symptoms, a greater proportion of ID Migraine+ participants reported that their FPP symptoms felt different than their headache symptoms compared to ID Migraine− participants (97.9% vs. 80.9%, *P* < .001). There were, however, no differences between the subgroups in the temporal relationship between headache and FPP symptoms. The ID Migraine− subgroup was significantly more likely to describe FPP symptoms as “always more severe” (15.6% vs 3.0%, *P* < .05 after Bonferroni adjustment) and “always lasting longer” (37.5% vs. 13.0%, *P* < .05 after Bonferroni adjustment) than their headache.

#### Disabling features

The ID Migraine+ subgroup reported greater rates of daily interference for any symptom in general (97.5% vs. 87.5%, *P* < .001), and specifically for headache (88.6% vs. 54.2%, *P* < .001) and dizziness (51.1% vs. 33.3%, *P* = .019) (Table 2). Participants in the ID Migraine+ subgroup most frequently reported headache as their most disabling symptom (74.1% vs. 42.9%, *P* < .05 after Bonferroni adjustment) followed by FPP (25.7% vs. 6.2%, *P* < .05 after Bonferroni adjustment), whereas a greater proportion of ID Migraine− participants reported otologic symptoms (5.7% vs. 0.5%, *P* < .05 after Bonferroni adjustment) as their most disabling symptom. Compared to ID Migraine− participants, a greater proportion of ID Migraine+ participants had a MIDAS grade of IV and higher median MIDAS score.

#### Allergies and sinusitis

The proportion of persons with allergies in the FPP+ group was 66% overall and did not vary significantly between ID Migraine subgroups, although the allergy testing rating was greater in the ID Migraine+ subgroup (76.5% vs. 51.7%, *P* = .004). There was no difference in the proportions with sinus infection or sinusitis (45.8% vs. 48.4%, *P* = .735). There were also no significant differences between groups for lifetime frequency of sinus infection, nor for antibiotic use, indication, effectiveness, frequency, or use duration.

### Headache and associated symptoms in FPP+ group's ID Migraine+ and ID Migraine− subgroups

To further explore differences between FPP+ ID Migraine subgroups for occurrence of headache in addition to photophobia, phonophobia, and nausea or vomiting, we examined composite variables. In the ID Migraine+ subgroup (*n* = 438), 302 (68.9%) participants had headache and photophobia. In the ID Migraine− subgroup (*n* = 48), 16 (33.3%) participants had headache and photophobia. The group difference of 35.6% was statistically significant (*P* < .001). Of those who had headache in the ID Migraine+ subgroup (*n* = 389), 302 (77.6%) had photophobia. Of those who had headache in the ID Migraine− subgroup (*n* = 39), 16 (41.0%) had photophobia. The subgroup difference of 36.6% was statistically significant (*P* < .001).

In the ID Migraine+ subgroup, 273 (62.3%) participants had headache and phonophobia. In the ID Migraine− subgroup, 19 (39.6%) participants had headache and phonophobia. The subgroup difference of 22.7% was statistically significant (*P* = .002). Of those who had headache in the ID Migraine+ subgroup, 273 (70.2%) had phonophobia. Of those who had headache in the ID Migraine− subgroup, 19 (48.7%) had phonophobia. The subgroup difference of 21.5% was statistically significant (*P* = .006).

In the ID Migraine+ subgroup, 213 (48.6%) participants had headache and nausea or vomiting. In the ID Migraine− subgroup, 8 (16.7%) participants had headache and nausea or vomiting. The subgroup difference of 31.9% was statistically significant (*P* < .001). Of those who had headache in the ID Migraine+ subgroup, 213 (54.8%) had nausea or vomiting. Of those who had headache in the ID Migraine− subgroup, 8 (20.5%) had nausea or vomiting. The subgroup difference of 34.3% was statistically significant (*P* < .001).

### Demographics, symptoms, disabling features, allergies and sinusitis in FPP+ group's ID Migraine− and FPP− group's ID Migraine+ subgroups

Results from the subgroup analysis examining demographics, symptom variables, disabling features, and allergies and sinusitis between FPP+ participants without migraine (*n* = 48) and ID Migraine+ participants without FPP (*n* = 85) are in Table 3.

**Table 3:** Patient demographic and symptom characteristics: FPP+/ID Migraine− (isolated FPP) vs. FPP−/ID Migraine+ (Isolated Migraine).

Variable	FPP+/ID Migraine – (*n* = 48)	FPP−/ID Migraine+ (*n* = 85)	Total (*n* = 133)	*P*
Demographics				
Age, yr (mean ± SD)	60.57 ± 11.46	51.18 ± 13.82	54.62 ± 13.73	<.001
Sex				.262
Female	40 (83.3)	77 (90.6)	117 (90.6)	
Male	8 (16.7)	7 (8.2)	15 (11.3)	
Undeclared	0 (0.0)	1 (1.2)	1 (0.8)	
Race				.755
American Indian or Alaska Native	2 (4.2)	3 (3.5)	5 (3.8)	
Asian	0 (0.0)	3 (3.5)	3 (2.3)	
Black or African American	2 (4.2)	2 (2.4)	4 (3.0)	
Native Hawaiian or Other Pacific Islander	0 (0.0)	1 (1.2)	1 (0.8)	
White	42 (87.5)	71 (83.5)	113 (85.0)	
Other	1 (2.1)	1 (1.2)	2 (1.5)	
Undeclared	1 (2.1)	4 (4.7)	5 (3.8)	
Ethnicity				.296
Non-Hispanic	48 (100.0)	81 (95.3)	129 (97.0)	
Hispanic	0 (0.0)	4 (4.7)	4 (3.0)	
Symptom variables				
History of frequent nasal symptoms	41 (85.4)	33 (38.8)	74 (55.6)	<.001
History of dizziness	38 (79.2)	73 (85.9)	111 (83.5)	.317
History of auditory symptoms	41 (85.4)	70 (82.4)	111 (83.5)	.648
Headache that limited activities for ≥1 day in last 3 mo	26 (54.2)	77 (90.6)	103 (77.4)	<.001
Headache symptom nausea/vomiting	1 (2.1)	67 (78.8)	68 (51.1)	<.001
Headache symptom light sensitivity	7 (14.6)	77 (90.6)	84 (63.2)	<.001
History of frequent or severe headache	48 (100.0)	85 (100.0)	133 (100.0)	–
Family history of migraine disorder or frequent headache	25 (52.1)	53 (62.4)	78 (58.6)	.248
Age when initial headache occurred, yr				.035
<12	4 (8.3)	23 (27.1)*	27 (20.3)	
12–25	22 (45.8)	37 (43.5)	59 (44.4)	
26–40	16 (33.3)	21 (24.7)	37 (27.8)	
41–65	6 (12.5)	4 (4.7)	10 (7.5)	
Nasal secretions description	*n* = 25	*n* = 18	*n* = 43	.563
Thin and clear	19 (76.0)	16 (88.9)	35 (81.4)	
Thick and discolored	3 (12.0)	1 (5.6)	4 (9.3)	
Thick and white	3 (12.0)	1 (5.6)	4 (9.3)	
Disabling features				
Symptoms interfere daily	42 (87.5)	81 (95.3)	123 (92.5)	.102
FPP symptoms interfere daily	22 (45.8)	4 (4.7)	26 (19.5)	<.001
Dizziness symptoms interfere daily	16 (33.3)	39 (45.9)	55 (41.4)	.158
Ear symptoms interfere daily	14 (29.2)	12 (14.1)	26 (19.5)	.036
Headache symptoms interfere daily	26 (54.2)	63 (74.1)	89 (66.9)	.019
Most disabling symptom				<.001
Dizziness	8 (22.9)	18 (26.1)	26 (25.0)	
Ear	2 (5.7)	3 (4.3)	5 (4.8)	
FPP	9 (25.7)*	0 (0.0)	9 (8.7)	
Headache	15 (42.9)	47 (68.1)*	62 (59.6)	
Unknown	1 (2.9)	1 (1.4)	2 (1.9)	
Allergies and sinusitis				
Allergies	30 (62.5)	35 (41.2)	65 (48.9)	.018
Allergy testing	*n* = 20	*n* = 35	*n* = 64	.665
	15 (51.7)	20 (57.1)	35 (54.7)	
Kind of allergy				
Trees, grass, weeds, pollen	13 (27.1)	13 (15.3)	26 (19.5)	.100
Animal dander	10 (20.8)	8 (9.4)	18 (13.5)	.064
Dust mites	11 (22.9)	13 (15.5)	24 (18.2)	.286
Mold	11 (22.9)	7 (8.2)	18 (13.5)	.017
Other	3 (6.3)	4 (4.7)	7 (5.3)	.702
Not sure	1 (2.1)	0 (0.0)	1 (0.8)	.361
Sinus infection of sinusitis	22 (45.8)	9 (10.6)	31 (23.3)	<.001
Sinus infection lifetime frequency	*n* = 20	*n* = 9	*n* = 31	.347
1–3	3 (13.6)	2 (22.2)	5 (16.1)	
4–6	5 (22.7)	2 (22.2)	7 (22.6)	
7–9	6 (27.3)	0 (0.0)	6 (19.4)	
>10	8 (36.4)	5 (55.6)	13 (41.9)	
Antibiotics				
Antibiotic use	*n* = 38	*n* = 67	*n* = 105	.577
	6 (15.8)	8 (11.9)	14 (13.3)	
Antibiotic indication	*n* = 4	*n* = 5	*n* = 9	1.00
Preventive	0 (0.0)	0 (0.0)	0 (0.0)	
Acute	4 (100.0)	4 (80.0)	8 (88.9)	
Other	0 (0.0)	1 (20.0)	1 (11.1)	
Antibiotic effectiveness	*n* = 5	*n* = 5	*n* = 10	.343
Not effective	1 (20.0)	2 (40.0)	3 (30.0)	
Minimally effective	0 (0.0)	1 (20.0)	1 (10.0)	
Moderately effective	2 (40.0)	0 (0.0)	2 (20.0)	
Extremely effective	2 (40.0)	2 (40.0)	4 (40.0)	
Antibiotic effective for FPP	*n* = 38	*n* = 67	*n* = 105	.037
	4 (10.5)	1 (1.5)	5 (4.8)	
Antibiotic frequency	*n* = 5	*n* = 4	*n* = 9	.358
Less than 1 day per month	3 (60.0)	4 (100.0)	7 (77.8)	
Less than 15 days per month	1 (20.0)	0 (0.0)	1 (11.1)	
More than 15 days per month	1 (20.0)	0 (0.0)	1 (11.1)	
Antibiotic duration	*n* = 5	*n* = 4	*n* = 9	.764
Less than 1 month	3 (60.0)	2 (50.0)	5 (55.6)	
More than 1 year	2 (40.0)	2 (50.0)	4 (44.4)	

Categorical variables presented as frequency (%). Denominators are depicted in the top row of the table unless otherwise shown within the variable row. Variables with multiple comparisons adjusted using Bonferroni correction.

* Depicts where statistical significance occurred after Bonferroni adjustment. Fisher's exact test is used for variables with cell counts less than 5.

#### Demographics

Participants in the FPP+/ID Migraine− subgroup were older, but there were no significant differences in sex, race, or ethnicity compared to the FPP−/ID Migraine+ subgroup.

#### Symptoms

In the FPP (FPP+/ID Migraine−) and migraine (FPP−/ID Migraine+) subgroups, 100% of participants reported history of frequent or severe headache. The proportion with initial headache below the age of 12 years was higher in the FPP−/ID Migraine+ subgroup. History of frequent nasal symptoms was more than twice as common in the FPP+/ID Migraine− subgroup (85.4% vs. 38.8%, *P* < .001), whereas the proportions reporting vestibular and otologic symptoms were elevated and similar in both subgroups. Headache severe enough to restrict activity or associated with nausea/vomiting or light sensitivity was higher in the FPP−/ID Migraine+ subgroup.

#### Disabling features

There were differences between the subgroups for the most disabling symptoms (*P* < .001), with a higher proportion reporting headache in the FPP−/ID Migraine+ subgroup (68.1% vs. 42.9%), and a higher proportion reporting FPP symptoms in the FPP+/ID Migraine− subgroup (25.7% vs. 0.0%).

#### Allergies and sinusitis

Participants in the FPP+/ID Migraine− subgroup were also more likely to report allergies (62.5% vs. 41.2%, *P* = .018), a history of sinus infection (45.8% vs. 10.6%, *P* < .001), and antibiotic effectiveness for FPP symptoms (10.5% vs. 1.5%, *P* = .037).

## Discussion

The premise on which this report is founded is that people with facial pain report that they consider this a symptom characteristically distinct from their headache. Among the FPP+ registrants, 97.9% (425/434) of migraine ID+ reported feeling that their headaches were different from their FPP. In contrast, 80.9% (38/47) who were Migraine ID- reported that same distinction. Our first aim, to investigate and describe the demographic and clinical characteristics of FPP that might implicate either migraine or RS as the underlying etiology, revealed a number of noteworthy findings. First and foremost, in this population the relationship between FPP and headache, and in particular migraine, was robust and multifaceted. Migraine diagnosis, based on a validated screening tool utilizing headache-associated restriction of activity, photophobia, and nausea and vomiting, was significantly more common in participants with FPP than those without. Our findings of an FPP demographic profile reminiscent of migraine (i.e., younger, more likely female), a higher proportion with family history of migraine or severe headache, and headache as the most common reason for disability may be related to the outsized proportion of migraine in this population, but it does not exclude the potential of shared hormonal and genetic mechanisms contributing to the pathophysiology of both migraine and FPP. The FPP group had a higher proportion of nasal symptoms commonly described with RS, as well as vestibular and otologic symptoms, which are decidedly less common in RS (20–22). These symptoms, however, may also be caused by the non-infectious inflammatory activation of autonomic and vestibular pathways that occurs in migraine (23–25), so are not precise in differentiating the conditions.

Responses to direct questions about alternative diagnoses, revealed that both allergies and history of sinusitis were more common in the FPP cohort, implicating a role of immune/inflammatory pathways in the connection between FPP and these disorders. We also identified an association of allergies and RS with FPP in a small-sample comparison of an FPP subgroup without migraine to a migraine subgroup without FPP. A recent Mendelian randomization study failed to demonstrate a causal relationship of allergic rhinitis and migraine, or the reverse (26). In light of this, findings from American Migraine Prevalence and Prevention study (27) showing that allergic rhinitis (coupled with a nonallergic rhinitis) was associated with higher migraine frequency and disability suggest that the epidemiological overlap may be related to shared mechanisms, a theory that requires further research (28). With regards to RS, our finding that antibiotics were more frequently prescribed in the FPP group, but were not effective, raises concern for misdiagnosis, which both delays proper treatment and contributes to the unnecessary use of antibiotics (29). Our findings of positive association of migraine and FPP coupled with the prominent overlap of symptoms between migraine and RS, should prompt early consideration of migraine and migraine screening, particularly when other causes of FPP have been ruled out by diagnostic evaluation with endoscopy and CT.

Our second aim, to examine demographics and clinical symptoms in persons with both FPP and migraine (compared to FFP without migraine), and in those where each condition occurs in isolation (FPP vs. migraine), revealed that in both subgroup comparisons those with migraine were younger, more likely to report early onset headache, and to describe headache as the predominant disabling symptom. Calling into question the role of migraine in the etiology of autonomic, vestibular and otologic symptoms, are our subgroup findings that (1) there were no significant differences in the prevalence of these three symptom constellations between the FPP subgroups with and without migraine, (2) there were no significant differences in the prevalence of vestibular and otologic symptoms in the FPP vs. migraine subgroup comparison, and (3) the prevalence of nasal/autonomic symptom in the isolated FPP subgroup was twice that of the isolated migraine subgroup. The etiology of these symptoms in migraine and FPP together and in isolation deserves further study in a population less enriched by headache. Neurogenic mechanisms may, however, have accounted for increased prevalence of facial pain over the forehead and eyes in persons from the FPP with migraine subgroup compared to the FPP subgroup without migraine, potentially by a migraine-related effect on different special sensory and autonomic neural networks. Our findings also raise the question of whether FPP and related symptoms may be neurogenically mediated as a variant of migraine without headache or migraine-defining associated symptoms.

### Limitations and future directions

There are a number of limitations in this exploratory study. Self-administered and self-reported surveys in general have the potential for recall bias, social desirability bias, and misinterpretation of questions. We aimed to minimize these biases through a number of measures: (1) limiting recall to 1 year; (2) including a “contact us” page on the registry site where participants can contact registry staff to ask questions; and (3) standardizing questions by using consistent terminology, sentence structure, and 6th grade level writing for easy comprehension.

There was also potential selection bias, with nearly half of participants coming to the registry from social media related to migraine. This likely accounted for the high prevalence of headache, and specifically migraine in our sample, and contributed to the finding that headache was the most common and most disabling symptom described by members in both groups (FPP+ and FPP−) and subgroups (ID Migraine+ and ID Migraine−, FPP+/ID Migraine− and FPP−/ID Migraine+). We will incorporate broader recruitment strategies beyond migraine groups to include individuals with head and neck symptoms without migraine. We also acknowledge that the sample sizes, especially for subgroup analyses, are small and likely not adequately powered to detect differences for many of the variables being compared.

Our preliminary findings indicate a lack of diversity in our sample with smaller proportions of men, Black, and Hispanic persons than found in the overall US population. We aim to ensure representation of the US population, and therefore the registry questionnaires are available in English and Spanish, however, additional recruitment strategies are necessary to engage with diverse communities by expanding the registry to include underserved healthcare settings (e.g., community health care centers and First Nations/indigenous communities).

Another limitation is missing data for some variables due to the fact that all questions have up to this point been optional, resulting in smaller sample sizes for some variables. To address this issue, we will require responses for all survey questions for future enrollees. In addition, a question on the throbbing or pulsating quality of headache pain, part of the International Classification of Headache Disorders 3rd edition criteria for migraine (16), was not included in the original questionnaire but will be included in the future version. Finally, given the cross-sectional nature of our preliminary findings, the relationship of any observed symptom or comorbidity to migraine pathophysiology remains speculative. A more robust sample with multiple data time points will also allow for more comprehensive statistical methods including multivariate analysis such as mixed effects modeling and regression analysis.

Further exploration of the relationship between FPP, rhinosinusitis and migraine will benefit from additional registry recruitment, including persons with rhinosinusitis, head, and neck symptoms, but no history of migraine, in addition to collection and documentation of objective clinical data, including neuroimaging, sinus computed tomography and endoscopy, as well as response to therapies prescribed for migraine, allergies and rhinosinusitis. Utilization of a more comprehensive, validated tool for diagnosis of migraine, its subsets and associated symptoms, as well as collection of a wide range of symptoms (e.g., autonomic, vestibular, and otologic) may be useful in capturing migraine's full spectrum of presentation. Such data will help fill a significant knowledge gap in the literature (30), which stems from the diagnostic difficulties regarding the etiology of facial pain and pressure symptoms that may include nasal congestion and rhinorrhea in the absence of any radiologic evidence of sinus infection or inflammation.

## Conclusion

This exploratory study of facial pain or pressure in a population from the HEADS Registry demonstrates an association of FPP with headache, including migraine, as well as with allergies, rhinosinusitis and related antibiotic use. The low rate of antibiotic effectiveness suggests misdiagnosis in at least a subset of those treated. Our finding of an association of FPP with autonomic, vestibular and otologic symptoms suggests neurogenic underpinnings, as distinct from an infectious etiology, but clinical characteristics may not be sufficient to make an accurate diagnosis. Uncovering these complexities and expanding the differential diagnosis to include conditions of neurological etiology, may lead to more accurate diagnostic and effective therapeutic approaches. This study has also revealed some of the limitations of the recently established HEADS registry, prompting addition and revision of questions, improvement in recruitment practices, and documentation of diagnostic studies, treatments, and outcomes. These preliminary findings and proceeding registry modifications will be key steps for the aim of ultimately using the HEADS Registry to improve outcomes for persons with neurologic, otologic and rhinologic conditions.

## Data Availability

The raw data supporting the conclusions of this article will be made available by the authors, without undue reservation.
