# Supplementary figures and images for: Facial/sinus pain or pressure and migraine: exploratory findings from the HEADS registry
Source: Front Pain Res (Lausanne). 2025 Aug 21;6:1625442. doi: 10.3389/fpain.2025.1625442 (PMC12408499; doi:10.3389/fpain.2025.1625442)

**Supplemental Material.** Data items from the HEADS Registry

**
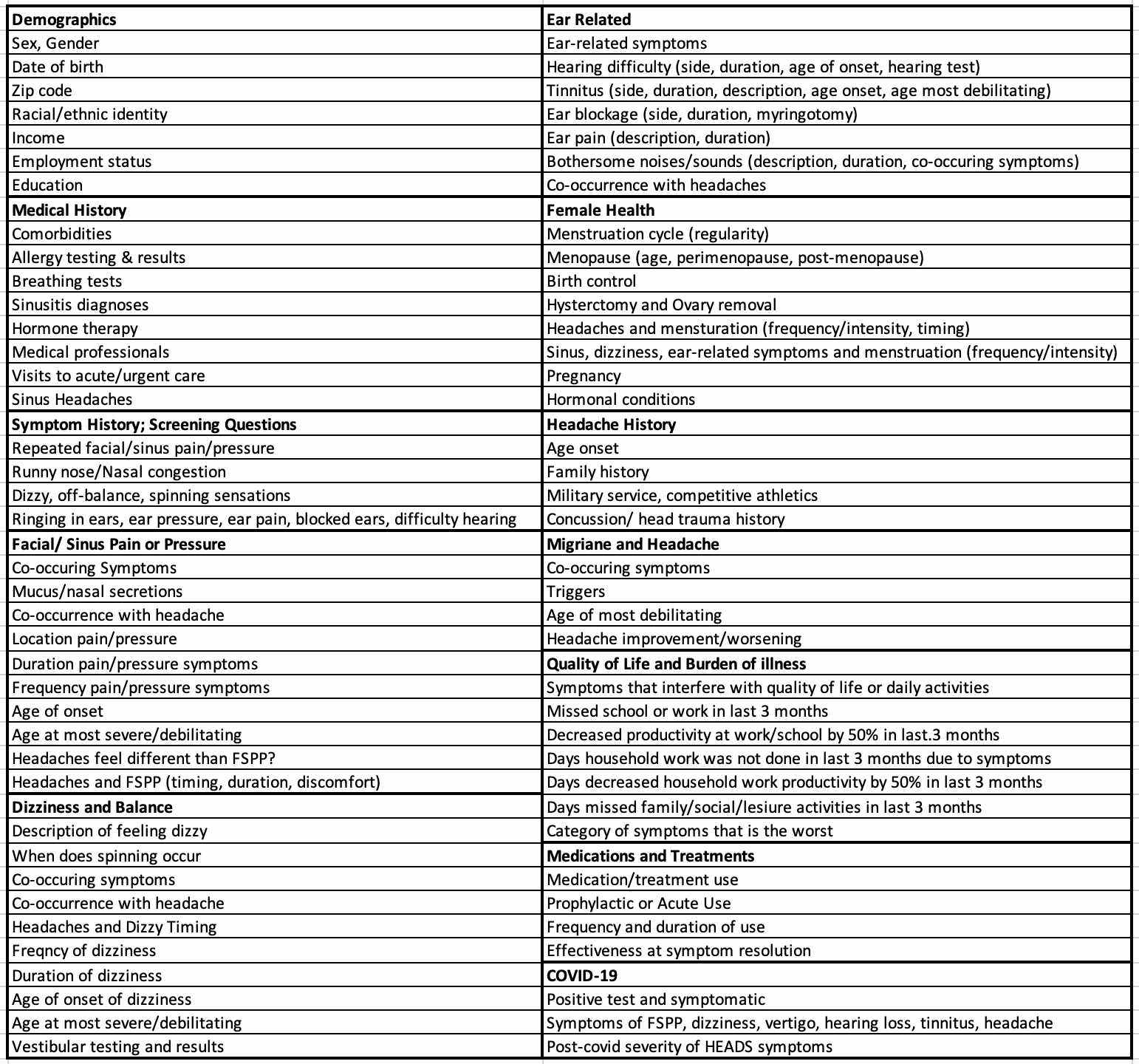
**

Supplement: Supplementary file 1 [file Table1.docx]
